# Introduction: Perception Without Representation

**DOI:** 10.1007/s11245-017-9460-1

**Published:** 2017-03-23

**Authors:** Roberta Locatelli, Keith A. Wilson

**Affiliations:** 1 Department of Philosophy, Philosophisches Seminar and Philosophy of Neuroscience Group, Centre for Integrative Neuroscience, University of Tübingen, Otfried-Müller-Str. 25, 72076 Tübingen, Germany; 2 Centre for the Study of Perceptual Experience, University of Glasgow, 69 Oakfield Avenue, Glasgow, G12 8QQ UK

## The Terms of the Debate

The idea that perceptual experiences have representational content has become something of an orthodoxy in recent philosophy of perception. It forms part of a view that aspires to extend some of Frege’s (1956) insights on thought to other mental occurrences such as beliefs, judgements, recollections and imaginings to provide a general and integrated account of the mind. The notion of representation also plays a central, some say ineliminable (Burge 2005, 2010), role in perceptual science, and for those who endorse computational theories of mind (e.g. Fodor 1975; Marr 1982). Nevertheless, some philosophers, including many of the contributors to this special issue, have sought to deny that perception is fundamentally representational. Such denials need not be taken to oppose current perceptual science. Rather, the claim—or at least one version of it—is that *conscious perceptual experience* is neither reducible to nor explicable in terms of representational states or content.^1^ In particular, advocates of non-representational views of perception maintain that the phenomenal character of veridical perception—broadly, “what it is like” (Nagel 1974) for the subject to undergo the relevant experience—is explained by the obtaining of a non-representational psychological relation to external mind-independent objects. This places the resulting views in opposition not only to representational or intentional theories, but also to adverbialism (Ducasse 1942; Chisholm 1957; Tye 1984), sense-datum theory (Broad 1952; Moore 1953), and non-representational qualia or ‘mental paint’ (Block 1996, 2010) views of perception.

In contrast to this representationalist orthodoxy, relational views characterise perceptual experience in terms of a primitive non-representational relation to external objects, facts or events. While both Naïve Realist^2^ and sense-datum theories claim that perception involves an irreducible relation to, or “acquaintance” (Russell 1912) with, objects, the theories differ with respect to the kinds of objects with which we are perceptually acquainted. According to Naïve Realism, these are the everyday external objects or events and their properties that we seem to perceive, where this may include perceptual ephemera such as rainbows, sounds or clouds. According to sense-datum theory, on the other hand, perception doesn’t relate us directly to these external objects, but to sense-data.^3^ Sense-datum theories, however, face distinctive problems of their own and have since largely fallen out of favour.^4^ We will therefore reserve the terms ‘relationalism’ and ‘relational views’ for the latter family of views which includes Naïve Realism (Martin 1997, 2004, 2006), Campbell’s ‘Relational View’ (2002), and Brewer’s ‘Object View’ (2011, this issue).

This special issue focuses upon the debate between representational views and these emerging relational views of perception. In particular, we aim to shed light upon the commitments and motivations of the latter which, being historically more recent and less widely held, have all too often been poorly understood by their detractors, many of whom have taken such views to be wildly implausible, incompatible with current scientific theory, or simply inscrutable. Indeed, for those steeped in the representational tradition, it can be difficult to understand why one might want to deny what may seem an obvious truth about perceptual experiences: that they represent how things in the world are. Furthermore, it is unclear that representational and relational views should be considered mutually exclusive, since relationalism need not be formulated in terms of the denial of representational content, and nor do these options exhaust the field.^5^ Indeed, some variants of the views have much in common (Sect. 2, 3).

We aim to elucidate relational views of perception in a way that facilitates a more nuanced debate (see Brewer, Travis, Martin). Other contributions explore the phenomenal character of experience and its explanatory role (Brogaard, Dokic and Martin, Eilan), and reappraise existing arguments both for (Brogaard) and against (O’Sullivan, Judge, Ivanov) relational views. We hope that this goes some way towards demonstrating that, far from being an implausible fringe view, relational theories constitute a significant and genuine attempt to overcome some central problems in the philosophy of perception, and, as such, are worthy of further consideration—not least by their opponents.

## Representational Views

The orthodox view of perception, which we will call *representationalism*, is that perception relates us to the world by representing it to be some particular way—for example, that some object instantiates specific properties. This makes experiences analogous to thoughts in that they have contents that are objectively, or possibly intersubjectively, evaluable for truth or accuracy. Such views have the benefit of unifying a diverse range of experiences, including hallucinations and illusions, with non-perceptual states such as thoughts, beliefs, desires, imaginings, recollections and intentional actions.

Representational theories of perception are often known as ‘intentional’ theories, not to be confused with *intentionalism*, which makes a stronger claim concerning phenomenal character (see below). The term ‘intentionality’ has its origins in scholasticism and was revived by Brentano (1874) and the phenomenological tradition.^6^ In its contemporary usage, intentionality is often held to be the distinctive mark of mental states and occurrences, indicating that they are directed towards, or about, worldly objects or states of affairs. In relation to perception, however, the label is potentially confusing since most if not all theories of perception take perceptual states to be directed towards or about the world in some sense. Representationalists, however, hold that perception is about the world in virtue of its representing a particular portion of it as being some specific way, or ways; e.g. blue, loud, sweet, and so on.

There has been much debate over the precise nature and structure of the content of experience, and how it compares to the content of belief.^7^ For example, some representationalists take perceptual content to be propositional (e.g. McDowell 1994; Brewer 1999), e.g. assessable for truth or falsity, while others take it to be, at least in part, pre- or non-conceptual (e.g. Peacocke 1998, 2001a, b; Kelly 2001; Bermudez 2009; Dretske 1969, 1981). However, it is generally taken to be a constraint upon representational theories of perception that such content has *veridicality, accuracy* or *correctness conditions*.^8^ Unlike philosophical notions of truth and falsity, which are standardly taken to be bivalent, these notions may admit of variations in degree. The mere existence of veridicality conditions, however, does not entail representationalism as we are defining it. For example, it is possible that experiences might possess veridicality conditions that are describable from some third-personal point of view, such as that of vision science, with the resulting content being entirely subpersonal. Subpersonal contents are not contents of any conscious experiential state or episode, and so do not qualify as contents of experience. A successful argument for representationalism must therefore show that the relevant accuracy conditions are in some sense available to, or accessible by, the subject at a first-personal level; e.g. in reasoning or action.^9^

In addition to their promise of offering an integrated account of the mind in terms of intentional states or processes, representational views are claimed to possess several significant advantages. First, they support a straightforward account of illusions and hallucinations which, like false beliefs, are held to have substantially the same content as veridical perceptions, but whose veridicality conditions are not met. That is to say, non-veridical experiences *misrepresent*. This is sometimes claimed (e.g. by Byrne 2009) to offer representational views a *prima facie* advantage in that they unify a diverse range of perceptual phenomena under a single *explanans*—namely, representational content. However, relationalists dispute that the principal *explananda* for a theory of perception include hallucinations and illusions, as opposed to focusing upon the central case of veridical perception. This methodological difference concerning the priority of veridical perception over non-veridical experience, as compared to the desire for a parsimonious explanation of both kinds of cases, is part of what motivates the present debate (Sect. 3).

Prior to the emergence of modern representationalism, the possibility of perceptually indistinguishable hallucinations and illusions had often been taken to support *indirect realism*; e.g. sense-datum theory (Moore 1918, 1926; Price 1932; Broad 1952; Russell 1921) or the representative realism of Locke (2008) or Hume (2007). This may be contrasted with *direct realist* accounts of experience according to which we are directly or immediately aware of external objects rather than via some perceptual intermediary, such as sense-data, ideas or impressions. Representationalism, however, claims to offer a simpler and allegedly superior explanation of the possibility of perceptually indistinguishable illusions and hallucinations, viz: subjectively indistinguishable non-veridical experience involves the representation of type-identical content to the equivalent veridical experience despite being caused in a non-standard or deviant manner. Thus, subjectively indistinguishable veridical and non-veridical experiences share a representational ‘common factor’ or content. Representationalists claim that this is compatible with direct realism since representational contents are not themselves *objects* of perception, as with sense-data, but part of the *means by which* we perceive objects. Whether this genuinely succeeds in securing direct realism, however, and whether, given representationalists’ other commitments, a unified account of perception and hallucination is available to them, are disputed.^10^

Second, representational views offer an appealing explanation of how experiences justify or rationalise perceptual judgements and beliefs. In the simplest case, experiences having personal-level contents makes it easy to explain how these can be “taken up”, “subscribed to” or “taken at face value” (McDowell 1994) in judgement and belief. More generally, the contents of perceptual beliefs may be held to be systematically related or identical to the contents of experience, thereby explaining how perception justifies or rationalises those beliefs. A parallel explanation can be given in the case of action. That beliefs and intentional actions have contents, however, is common ground between representational and non-representational views of experience, which differ only in respect of whether *experiences* have contents and so are themselves belief-like. This places an explanatory burden upon the anti-representationalist to explain how experiences can justify or rationalise beliefs if not via their contents. A comparable explanatory burden, however, falls upon the representationalist to explain what fixes or individuates contents of experience, and how this can justify beliefs even if, according to common factor views, it is possible for non-veridical experiences to have type-identical contents. Determination of content is therefore an issue for both views, though for the anti-relationalist this relates to the contents of perceptual judgements or beliefs rather than experiences (see Sect. 3).

Related to the common factor view described above, and central to the dispute with relationalists, a further claim made by many representationalists is that the phenomenal character of an experience is determined by its representational content and/or manner of representation (e.g. blurriness). This view, known as *intentionalism*, or sometimes simply ‘representationalism’,^11^ admits of both weak and strong variants. According to *weak intentionalism*, an experience’s phenomenal character supervenes upon its representational content such that there can be no difference in phenomenal character without a difference in representational content.^12^ According to *strong intentionalism*, on the other hand, phenomenal character is identical to at least some elements of an experience’s content; e.g. its conceptual or non-conceptual content (cf. Tye 2002, 2007; Schellenberg 2011). As such, strong intentionalism entails weak intentionalism, but not vice versa. In both cases, however, it remains an important question whether the same, i.e. type-identical, content can consistently perform both epistemological and phenomenological roles—a point that some relationalists have disputed (Travis 2004, 2013a).

In summary, representational views take many different forms, each reflecting the various roles that perceptual representation might play: individuating experiences, explaining or justifying the contents of beliefs, grounding the qualitative character of experience, and explaining the causal mechanisms involved in perceptual processing, to name but a few. Consequently, there are a wide variety of views that differ both in strength and in the kind of contents that they assign to perceptual states or episodes. These range from the mere attribution of representational content to perceptual states without any commitment to this constituting a fundamental characterisation of experience—a view sometimes known as the “weak content view” (Brogaard 2014a: 2)—to exhaustively characterising experiences and/or their phenomenal character in representational terms, as per representationalism and intentionalism, respectively. Additionally, each view may be held in conjunction with the attribution of non-conceptual and/or non-propositional contents, be these particular or general, that are held to play a variety of explanatory roles.

Despite frequently, and in our view somewhat misleadingly, being portrayed as a two-horse race, many (though not all) of the above views appear *prima facie* compatible with at least some variants of relationalism, yielding the possibility of hybrid views. For example, one might agree that the phenomenal character of perception constitutively involves external objects while holding that experiences nevertheless possess, or may be associated with, representational contents.^13^ For this reason, positions on opposite sides of the debate—e.g. Naïve Realism or relationalism (Martin 1997, 2004, 2006; Campbell 2002; Brewer 2011; Fish 2009; Soteriou 2013) and phenomenal externalism (Tye 1995; Lycan 2001)—can appear to have more in common than that which separates them, raising questions as to precisely what is at issue in the debate and how it is to be adjudicated. Moreover, as we have seen, representationalism does not preclude that perception directly relates us to perceptual objects. Rather, it purports to uphold a form of direct realism according to which we are ‘directly’ or ‘immediately’ aware of external objects, rather than via some perceptual intermediary such as sense-data (see Sect. 4). Indeed, representation is itself a kind of relation, one term of which is the material object, or its properties, that we seem to perceive.^14^ Furthermore, even those who deny that perceptual representation entails the existence of a perceptual relation (e.g. Crane 2006) agree that we are causally related to perceived objects. If relationalism is to be distinct from representationalism, then it needs to involve a more specific claim.

## Relational Views

Relational theories of perception take veridical experience to involve a primitive, and hence unanalysable, metaphysical relation to external objects and their properties. While variants of the view differ as to precisely which objects or properties, facts or events, are admissible as relata of experience, they agree that the relational nature of perception contributes to, or determines, the phenomenal character of experience. Thus, while characterising perception in relational terms might appear to leave it open whether experiences also possess representational contents, in practice many relationalists oppose such content on the grounds that it fails to adequately characterise the fundamental nature of veridical perception (Brewer 2011, this issue), or that it is explanatorily redundant (Travis 2004, 2013a). These stronger forms of relationalism thus oppose both intentionalism and representationalism (see Brogaard this issue; Travis this issue), and are the target for recent defences of those views (e.g. Byrne 2009; Burge 2010; Schellenberg 2011). The dispute between representationalists and relationalists therefore is as much concerned with what plays a particular explanatory role, i.e. determining the phenomenal character of experience, as it is the metaphysics of experience (Wilson forthcoming).

Relationalists are motivated by a variety of methodological, phenomenological and epistemological considerations. An illustrative, though non-exhaustive, list includes explaining:

i. The transparency of experience in a way that is least revisionary with respect to how experience strikes us introspectively (Martin 2002)
ii. The primacy of external objects and/or veridical perception in an overall theory of perception (Brewer 2011)
iii. The relationality of perception in a way that avoids positing metaphysically problematic entities such as sense-data (*ibid*.)
iv. The possibility of demonstrative knowledge and/or reference (Campbell 2002)
v. The possibility of thought about the external world as compared to the direct presentation of particulars (Travis 2007, 2012, 2013b)
vi. Epistemic humility concerning judgments about the kind of experience that one is having (Martin 2004, 2006).^15^

Precisely what relationalists mean by the ‘fundamental nature’ of perception, and how the relationality of experience determines phenomenal character, however, require further explication. In relation to the latter, John Campbell writes

On a Relational View, the phenomenal character of your experience as you look around the room, is constituted by the actual layout of the room itself: which particular objects are there, their intrinsic properties, such as colour and shape, and how they are arranged in relation to one another and to you. (2002: 116)

Similarly, for M. G. F. Martin:

According to [Naïve Realism], the actual objects of perception … partly constitute one’s conscious experience, and hence determine the phenomenal character of one’s experience. (2004: 93).

For such relationalists, the phenomenal character of perception is partly constituted by the objects that one experiences and their perceptible properties. Unfortunately, it is not always clear what relationalists mean by this claim. One way of understanding it, however, follows from the nature of the perceptual relation, which cannot obtain without the presence of its external relatum: the object(s) of perception. Consequently, external objects are metaphysically constitutive of both experience and its phenomenal character.^16^ This constitution claim renders relationalism *prima facie* incompatible with intentionalism, which offers a purely representational explanation of phenomenal character (Sect. 2), while leaving it open whether experiences have representational contents that fulfil some other explanatory role; e.g. in relation to perceptual knowledge. Furthermore, some externalist forms of representationalism on which an experience cannot have the same particular content in the absence of the object represented (e.g. Burge 1991; Dretske 1995; Lycan 2001; Schellenberg 2010) might be seen as compatible with this form of relationalism and the above interpretation of the constitution claim. This highlights the similarity between variants of each view, illustrating how difficult it is to precisely delineate the disagreement between relationalist and representationalist positions.

Given the central role that relationalists assign to veridical perception, however, two of the most important challenges for the view concern how to account for perceptual illusions and hallucinations, respectively. Concerning the former, Brewer claims that

[a]ny errors in her world-view which result are products of the subject’s responses to this experience, however automatic, natural or understandable in retrospect these responses may be. *Error*, strictly speaking, given how the world actually is, is never an essential feature of experience itself. (2006: 169)

Instead, the erroneous nature of perceptual illusions is explained in terms of “misinterpretation” (*ibid*.) or “misleading perception” (Travis 2004, 2013a), such as when a subject mistakenly judges that some perceived object instantiates a property, or properties, that it in fact does not possess. Whether these explanations amount to the same thing, and whether anti-representationalists need to posit some additional stage of perceptual interpretation or “seeming” (Brogaard this issue) that falls between perceptual experience and judgement, remain open questions. Nevertheless, as with the standard representationalist explanation of illusions, the metaphysical and explanatory structure of such experiences is consistent with that of the veridical case—a point that is sometimes overlooked by opponents of the view.^17^

Hallucinations, on the other hand, are more challenging for relationalism, since these are precisely cases in which the subject is not directly related to any causally relevant external object. To explain such cases, relationalists typically adopt some form of *disjunctivism* (Soteriou 2014, 2016). Indeed, if relationalism is characterised as a claim about what explains or constitutes the phenomenal character of experience, then it is already committed to a specific form of disjunctivism: namely, that the subjective character of veridical perceptions and hallucinations cannot be explained in the same way.^18^ In offering a positive account of hallucination whereby, for example, such experiences present uninstantiated universals (Johnston 2004), sense-data or intentional objects,^19^ the relationalist leaves it open that the same explanation also applies in the case of veridical perception. In the case of causally matching hallucinations that are brought about by the same brain state as a veridical perception, this gives rise to what Martin (2004, 2006) calls the “screening-off” problem, whereby it seems that whatever accounts for the phenomenal character of the hallucination should also suffice to explain the phenomenal character of a mental state brought about by the same brain state, thereby rendering redundant the explanatory power that relationalists attribute to the object of perception in the veridical case. To avoid this, some relationalists, including Martin and Brewer, endorse a negative form of disjunctivism according to which the only positive definition of the mental properties of causally matching hallucinations that can be given is that they are subjectively indistinguishable, or “indiscriminable”, from veridical perception (*ibid*.). Others find the lack of a positive characterisation of hallucination unsatisfying.

It is important to realise that many disjunctivists identify an experience’s phenomenal character with the obtaining of a relation to the objects and properties that, on their view, constitute it. On this understanding of the term, an experience’s phenomenal character is individuated more finely than what can be distinguished from a first-personal perspective. Hence two subjectively indistinguishable experiences can possess different phenomenal characters in virtue of the fact that they involve the perception of distinct objects and/or properties. Moreover, since hallucinations do not involve direct acquaintance with the object, then strictly speaking, they lack perceptual phenomenal character in this sense of the term. This does not of course mean that such disjunctivists deny there is anything it is like to undergo a hallucination. Rather, they claim that the subjective character of hallucination and that of veridical perception do not share the same constitutive nature. In other words, perceptual appearances, or looks, are multiply realisable. It is therefore no objection to relational views that they fail to unify veridical and non-veridical experiences. Rather, despite the possibility of subjective indistinguishability, relationalism involves precisely the claim that hallucinations cannot be, and indeed *should not* be, explained in the same way as veridical perception. Hence relationalists take the primary explanandum of a theory of perception to be veridical perception, and not perceptual experience more generally where this includes both veridical and non-veridical experiences, since the latter may require a different explanation that is dependent upon the former.

## ‘Direct’ Versus ‘Indirect’ Perception

According to sense-datum theories of perception, the immediate or ‘direct’ objects of perception are not everyday external objects, as in Naïve Realism, but a perceptual intermediary, or *sense-datum*. This yields a form of indirect realism which, according to Berkeley (1982), creates an unacceptable ‘veil of perception’ between the mind and the external world. Similarly, if perceptual representations, rather than the objects and properties that they represent, constituted direct objects of perceptual experience—a view often attributed to Locke—then the resulting theory would be perceptually indirect. In a move reminiscent of adverbialism,^20^ however, modern representationalists aim to avoid this objection by arguing that representations are not themselves *objects* of awareness, but part of the *means by which* we perceive such objects. As such, it is the external objects that experiences represent as having certain properties which should be regarded as direct objects of perception, and not their representational contents.^21^ Indeed, many representationalists, and therefore intentionalists, reject the idea that experiences strictly speaking possess an act–object structure, instead characterising them as merely causally or contingently related to the external objects of perception.

### Bill Brewer: The Object View

In his contribution to this issue, Bill Brewer responds to various objections addressed to the version of relationalism he proposes: the “Object View”. Among these is Brogaard’s (2014b) charge that, with its insistence on the contribution of the subject’s point of view and the conditions of perception in determining the phenomenal character of experience, the Object View can be assimilated to a position “on which our perceptual relation with *indirect* external objects of perception is somehow the result of computations on more basic direct objects such as retinal images” (this issue). Brewer responds that “there is no obvious reason to believe that every theory that gives the perceiver’s point of view a role in accounting for the way things look is committed to regarding such retinal images as the direct objects of perception in any sense” (*ibid*.). Thus, for Brewer, while computations on retinal images are “essential psychological enabling conditions” of our perceptual relation to external objects, they are not themselves objects of perceptual awareness. (See Sect. 5 for discussion of Brewer’s view of perceptual appearances, or looks.)

### Charles Travis

In ‘Deliverances (Indirection)’, Charles Travis addresses the question of whether representationalists are entitled to claim that perception furnishes us with a direct awareness of, or access to, environmental particulars, or whether representational content is itself problematically opaque. Framed as a continuation of his long-running debate with John McDowell (2008, 2013, ms), who endorses a disjunctive form of representationalism, Travis’s paper can also be read as offering a general critique of representationalists’ appropriation of the language of direct realism despite positing the existence of a theoretical intermediary—namely, representational content.

Travis begins by differentiating two kinds of ‘veiling’ that representational content might generate concerning the notions of *resemblance* and *instancing*, respectively. The former is a problem for Locke, who held that perceptual representations ‘resemble’ what they represent. However, since these representations, which are presumably grounded in physical properties of the brain, do not instantiate the perceptible properties that they attribute — roundness, blueness, loudness, and so on — “[w]e are left with no grasp of what resemblance in [Locke’s] sense might be” (Travis, this issue). Indeed, for Travis, Locke’s resemblance relation is simply ungraspable and, as such, cannot confer knowledge of how things in the world are.

The problem of instancing, on the other hand, is targeted at McDowell. If perceptual experiences represent how things in the world are, then in order to be of any epistemic use, it must be possible for us to grasp or recognise “*what* ways an experience represents things as being” (this issue; cf. Travis 2004, 2013a). According to Travis, experience must facilitate a passage from awareness of environmental particulars, i.e. external or what Travis calls “historical” objects and their properties, to awareness *that* something is the case, which is factive or “conceptual”.^22^ However, and herein lies the alleged problem, perceptual representation involves an awareness of generalities and not only particulars, and so already lies on the far side of this passage. Thus, Travis argues, either (a) representation cannot facilitate such a passage, and so is redundant, or else (b) it does so mysteriously, since nothing in the relevant representations can tell us what would count as instances of their contents. Hence, by analogy with Locke, the external world is “veiled” from us by the opacity of the instancing relation, which is neither given by, nor deducible from, perceptual experience on McDowell’s representational view.

Travis holds that experience merely *presents* external objects and their particular properties to the perceiver, without them being presented, or represented, as being any particular way. He argues that if content is to be epistemically useful, perception must at some point bottom out in such an awareness of environmental particulars prior to the tokening of content. For Travis, content does not enter the picture until the subject exercises their capacity for judgement or belief-formation. Travis and McDowell thus disagree over whether these content-tokening capacities are operative at the level of experience (McDowell’s view) or are post-perceptual (Travis’s view), with consequences for whether the resulting content is attributable to perceptual experiences or judgements, respectively. Here, Travis identifies a problem that is common to him and his opponents: explaining how the contents of perceptual judgements, if not of experiences proper, arise. The demarcation of perceptual and cognitive states, along with the nature and functioning of our recognitional and conceptual capacities, are thus central to this debate, the Fregean underpinnings of which are helpfully summarised in the final sections of Travis’s paper.

### M. G. F. Martin

While Travis and Brewer discuss where intentionalism and Naïve Realism sit with respect to the traditional debate about direct versus indirect realism, M. G. F. Martin sees the intentional approach as addressing the same concerns that were central to that debate, i.e. how objects determine the phenomenal character of experience. However, according to Martin, intentionalism does so in a way that widens the debate and renders the old dispute about the ‘direct object’ of perception outdated. With the introduction of the intentional framework, “the question now is not only which objects can feature in our experience, but how they can so feature” (this issue). Although the terms of the traditional debate are narrower than they need to be and “it might be proper to retire talk of direct perception and direct realism”, Martin argues, contrary to Austin (1962), that the traditional debate over direct realism is not empty or ill-defined, nor it is obvious or trivial in the way that Chisholm (1957) and others have contended. Instead, Martin proposes an illuminating reading of the significance of this debate, focusing on ‘Seeing Surfaces and Physical Objects’ in which Thompson Clarke (1965) takes issue with G. E. Moore’s claim that we only see the surfaces of objects. Although Clarke ultimately rejects Moore’s claim, he maintains that the question is serious and not trivial. If it turned out that, when seeing a tomato, we can only directly see its facing surfaces, we would hardly be better off than in a case where a portion of the tomato were sliced off and occluded the rest of it. We would not be related to the tomato in virtue of being related to the surface; we would be cut off from it.

The opposition between direct and indirect objects of perception is substantial only if we find a connotation of ‘object’ that “appl[ies] to some, but not all, ways of exemplifying it”. Unfortunately, neither Moore nor Clarke are explicit about how this opposition should be understood, and more explicit distinctions, such as Jackson’s (1977) “mediate” and “immediate” objects of vision, are uninformative. The relevant connotation may be clarified, however, with the help of an analogy with immediate versus mediate location. “Objects compete with each other for being immediately located in a region”, even if one is located in one region in virtue of being located in another. Similarly, the surface of the tomato seems to compete with the entire tomato in “fixing the overall look of the scene” and thus, according to Moore, in counting as the direct object of perception. In this competition, there are good reasons to think that the surface pre-empts the entire object, as the surface seems sufficient to fix the same look. This argument mirrors Martin’s own “screening off” argument (2004, 2006) that motivates his negative disjunctivism (Sect. 3). However, there is a solution available here that was not available with respect to the “screening off” problem: one can deny that seeing a surface pre-empts seeing the object. Although seeing a surface removed from its object and seeing the whole object might be subjectively indistinguishable from the point of view of the subject, they are indeed different situations, with different objects playing analogous roles in determining the phenomenal character of the experience.

Clarke, on the other hand, concedes that seeing the surface of the tomato pre-empts seeing the whole tomato in determining the phenomenal character of the experience. Thus the only strategy that remains available to him to defend the claim that we do, under normal circumstances, directly see the whole tomato is to commit to the indeterminacy of facts about experience. He maintains that the claim that we directly see only the surfaces becomes true only in special circumstances where we restrict the application of the verb ‘see’. This shows how difficult it is to resist Moore’s conclusion once we accept that “there is nothing more to what we know of our sense experience other than what we can report in terms of what we see”. If one wants to avoid Clarke’s commitment to the indeterminacy of perceptual facts, one ought to provide an account that accommodates our initial intuition that, when we see the tomato, we do not see only its surface.

 Unlike sense-datum theories, intentional views are conservative with respect to the question of what objects we are aware of when we perceive. They make space for this by being revisionary with respect to the way in which objects are presented in perception. It is not clear that this is in accord with our initial intuitions about our experience of objects. Thus, whether intentionalists can rightly claim that their account preserves direct realism will depend on whether the introduction of representational content “leaves unchallenged what we all can initially claim about our experience”.

## Appearances and the Phenomenology of Experience

As we have characterised them, relationalism and intentionalism purportedly offer competing accounts of the phenomenal character of experience (Sects. 2 and 3). Much of the debate between them therefore turns on the adequacy of these accounts. Here we focus on four such issues concerning the duality of perceptual appearances (Brogaard), the relational account of perceptual appearances and illusions (Brewer), the relevance of conscious experience for the possibility of thought (Eilan), and the sense of presence (Dokic & Martin), respectively.

### Berit Brogaard

In ‘The Silence of the Senses’, Charles Travis (2004, 2013a) argues that perceptual experiences cannot have representational contents because they are, in an important sense, equivocal or indeterminate between multiple ways that the world might be. Thus, in what one of us has elsewhere called Travis’s “*argument from looks*” (Wilson forthcoming), “in perception, things are not presented, or represented, to us as being thus and so. They are just presented to us, full stop” (Travis 2004: 65).^23^ Travis’s argument for this point draws on the various kinds of looks—comparative, epistemic, phenomenal—that might conceivably make such representations available to the subject, and concludes that none is able to do so. Thus, even if there were such a thing as perceptual representation, it would be cognitively inaccessible to the subject and so explanatorily redundant.^24^

Berit Brogaard (this issue) offers a new line of response to Travis.^25^ While Brogaard agrees with Travis that representation is not an essential feature of perceptual experience, she argues that empirical evidence concerning the duality of appearances favours the view that, for perceivers like us, visual experiences do represent the world as being some particular way, and so are not equivocal in the manner that Travis suggests.

Brogaard takes Travis’s argument to concern the independence of perceptual content from the agent’s epistemic states: beliefs, judgements, and so on. This is what we would expect if, as per representationalist orthodoxy, the contents of some such states—for example, the belief that some particular tomato is red—derive from the contents of experience, e.g. seeing the red tomato, rather than the other way around.^26^ If, in addition to this, the contents of experiences were themselves dependent upon the contents of beliefs, then the relationship between the two would be circular, and so not explanatory. Brogaard argues, however, that Travis’s denial that experiences have any recognisable content, or “face value”, fails to do justice to visual phenomenology. According to Brogaard, visual experiences can and do favour one interpretation over others, and so things can look to be a particular way independently of the subject’s epistemic states. Consequently, Brogaard argues, a key premise of Travis’s argument is false, and intentionalism is again off the hook.

Brogaard concludes by offering a positive “Argument from Phenomenology” in favour of representational content. Like Susanna Schellenberg’s “Master Argument” (2011), Brogaard argues that we cannot fully explain visual phenomenal character without appealing to the notion of “perceptual seeming”. According to Brogaard, such “seemings” favour intentionalism over non-representational alternatives. Of course, anti-representationalists might in turn dispute whether “seemings” are wholly perceptual as opposed to being complex, partly epistemic states, as with Brewer’s ‘thick’ notion of looks (Sect. 5.2). Nevertheless, since the phenomena Brogaard describe involve appearances that are robustly independent of the subject’s beliefs, in order to avoid her conclusion the anti-representationalist must either (a) posit the existence of some further non-doxastic state that falls between ‘pure’ experience and belief, or else (b) provide an analysis of such phenomena in terms of existing epistemic states. Brogaard’s Argument from Phenomenology can therefore be seen as challenging the adequacy of relationalism in accommodating the human visual system’s adherence to certain perceptual principles.

### Bill Brewer: Thin and Thick Looks

According to Brewer’s ‘Object View’ (Sect. 4.1), an object’s visually appearing or “thinly” looking *F* is explained by its possessing “appropriate *visually relevant similarities* with paradigm exemplars of *F*” (this issue), i.e. objects that are paradigmatically *F*.^27^ Thus, the lines of the Müller-Lyer illusion, for example, look to be of different lengths not because we represent them to be such, but because the presence of the arrowheads causes them to visually resemble paradigm cases of three-dimensional convex and concave edges that are closer to or further away from the subject, respectively (Brewer 2008). Due to the phenomenon of size constancy, our visual system automatically adjusts for such depth cues, causing the lines to appear as being unequal in length, as would in fact be the case in the equivalent three-dimensional figure. Despite their dependence upon contingent properties of our visual systems, such similarities are nevertheless mind-independent in that they are grounded in objective features of external objects independently of the subject’s thinking or representing anything to be so. Indeed, Brewer argues that such illusions are more problematic for orthodox representational views, which are committed to there being some fact of the matter as to precisely how long subjects represent the lines as being (*ibid*.).

Thin looks alone, however, are insufficient to explain the phenomenology of figures like the Necker cube or duck-rabbit which appear to flip between different possible appearances. Nor do they explain why we tend to register some of an object’s similarities over others. A white piece of chalk illuminated by red light, for example, possesses visually relevant similarities to both (a) red objects under normal illumination conditions, and (b) white objects illuminated by red light. Yet, even given that looking white-in-red-light is a way of looking white, we would not ordinarily say that white chalk in red light “looks white”, except possibly in Chisholm’s (1957) epistemic use of ‘looks’. To overcome these objections, Brewer appeals to a “thick” notion of looks which involves the “perceptual registration” of a particular visual similarity or appearance. Thus, in respect of its colour, the chalk *thinly* looks both red and white-in-red-light, but *thickly* only looks red, since this is the property that our visual systems typically register under normal circumstances. Brewer takes the central cases of perceptual registration to involve the application of a concept, but allows that there may be forms of registration, and so thick looks, that are non-conceptual. Interestingly, both of these cases can also be described in terms of the tokening of representational elements. Thus, Brewer’s theory accords with Brogaard in allowing a role for representational content in determining the phenomenal character of experience in the thick sense. Brewer argues, however, that this does not undermine the Object View’s claim to be a form of relationalism, since (a) it fundamentally characterises experience in terms of the subject’s relation to external mind-independent objects, and so as non-reducible to its representational content, and (b) the representational elements in thick looks are themselves dependent upon the obtaining of this perceptual relation.

### Naomi Eilan

In *Reference and Consciousness*, John Campbell (2002) argues that only a relational view of perception can adequately make sense of how conscious experience plays a crucial role in enabling demonstrative thoughts about the categorical properties and mind-independent objects that we perceive. According to relationalism, one is confronted with the very mind-independent objects that demonstrative judgments refer to, thereby explaining how perception can provide knowledge of the semantic values of such demonstratives. Conversely, Campbell argues that representational views fall short of accounting for the explanatory role of experience because by postulating conceptual content they presuppose what is to be explained: the possession and ability to use demonstrative concepts.^28^ To counter this argument, a representationalist could either (a) deny that experience plays the explanatory role that Campbell assigns to it (Burge 2005; Cassam 2011), or (b) argue that representationalism succeeds in explaining the role of experience in allowing us to have a conception of objects in the world as mind-independent, and of properties as categorical. Indeed, many representationalists, and in particular proponents of phenomenal externalism (cf. Tye 1995; Lycan 2001), would argue that the phenomenal character of experience plays precisely such a role in explaining the objective import of experience through its representational content.

Naomi Eilan (this issue) challenges both of the above strategies. She begins by formulating a challenge to representationalism on Burge’s behalf, which she labels “Burge*’s Challenge”. It takes the form of the following dilemma: either (i) endorse the general structure of Burge’s representational account of perception, which she labels “Caused Representation”, and give up on a role for consciousness, or (ii) relinquish Caused Representation, and defend a role for consciousness. As noted above, Burge himself opts for the first horn. On this account, everything there is to say about how perceptions achieve objective import also applies to blindsight (Weiskrantz 1986), which is often described as a case in which the subject lacks conscious experience despite enjoying a perception that accurately represents the properties of the external object.

The alternatives are to opt for the second horn, or to reject the dilemma. Many defenders of representationalism about perceptual experience would argue for the latter option: to give consciousness a role within a representationalist framework. To test this, Eilan formulates a sceptical challenge for claims to the effect that perceptions have objective import, which is to explain what makes it the case that we perceive particular categorical properties rather than their structural equivalents. She argues that it can be met only by relational adoptions of the second horn of the dilemma, and in particular by those versions that appeal to Russellian acquaintance with mind-independent objects and their properties, where such acquaintance is conceived of as “knowledge of things” independently of “knowledge of truths”.

The paper ends with a comparison between Eilan’s position and that adopted by phenomenal intentionalists (Horgan et al. 2004; Kriegel and Horgan 2008), who would reject the dilemma by endorsing Burge’s approach to the representational content of perception while at the same time insisting that consciousness is integral to perceptual forms of representation. Eilan argues that such accounts cannot meet the sceptical challenge in a way that, contra Burge and according to our intuitions, gives consciousness a role in delivering objective import.

### Jérôme Dokic and Jean-Rémy Martin

Another crucial aspect of the phenomenology of experience is the sense of reality, also known as a sense or feeling of presence. Against philosophers who see this sense of presence as constitutive of genuine perception, Jérôme Dokic and Jean-Rémy Martin argue that it is two-way independent from the contents of perception, and that this has an important implication for the debate over intentionalism and relationalism which results in intentionalism being unable to account for the sense of reality.

Dokic and Martin distinguish between two different phenomena that are commonly referred to by the expression “sense of presence”: (i) the sense that an object is real, or “sense of reality”, which is the central concern of their paper; and (ii) the sense that we are acquainted with the object itself rather than some surrogate or representation, which they call “sense of acquaintance”. To distinguish these, the authors consider studies on *derealisation*, a disorder in which, they argue, patients are best described as lacking the sense that what they experience is real while preserving otherwise normal perceptual capacities. This suggests that the sense of reality is not constitutive of perception. Rather, it is a specific experience that is “enjoyed over and above the perceptual experience itself”, and which might fail independently of other perceptual capacities.

While the case of derealisation shows that experience can occur without a sense of reality, three other cases show that derealisation may occur without a genuine experience: (i) an experience, common in cases of Parkinson’s disease, in which patients report that they vividly perceive the presence of a person without seeing, touching, hearing, or smelling that person, who is not in fact there; (ii) recent studies on virtual reality suggest that increasing the realism of spatiotemporal content doesn’t correlate with an increased sense of reality, nor vice versa; (iii) certain forms of hallucination where quite unrealistic entities are experienced as part of the world, presenting a similar dissociation of sense of reality from level of realism of the content of experience. Such cases suggest that the sense that an object is present does not require that the object is perceived.

Further to these empirical considerations, Dokic and Martin provide theoretical consideration for the claim that the sense of reality is not constitutive of perceptual experience. They suggest that the best explanation for this phenomenon is that it is an affective component, akin to a metacognitive feeling, “based on various *reality-monitoring* processes, and processes that control one’s spontaneous judgment of reality” (this issue). With this understanding of the sense of reality in place, the authors formulate the following challenge for intentionalism. Since genuine perception is possible without the sense of reality, how should perceptual experience be characterised in contrast to other kinds of sensory mental states, such as imaginings, which are clearly representational? The natural way for the intentionalist to do this would be to claim that, contrary to these other states, the content of perception asserts that the representation is veridical. But this conflicts with the above empirical and theoretical considerations that speak in favour of their independence. The alternative would be to deny that perception is fundamentally different from phenomena such as imagination, where the only distinction between the two is that the former, but not the latter, is accompanied by the distinctive sense of presence. According to Dokic and Martin, relationalism, on the other hand, “seems to be able to explain the specificity of perception independently of the instantiation of the sense of reality. Unlike imagination, perception is a relation to the world, and the veridicality of perceptual experience does not rest on the truth or correctness of a representation” (*ibid*.).

## Assessing Arguments for Representational Content

### Michael O’Sullivan

In addition to the kind of general philosophical considerations that Travis, Brogaard and others identify, a variety of empirical phenomena have been argued to count in favour of perceptual representation. Here, Michael O’Sullivan examines one such case from the psychological literature concerning the perception of numerosity.

In visual perception, arrays of objects that are too numerous to subitize, i.e. immediately recognise or ‘see’ their quantity, appear perceptually distinct from arrays containing different numbers of objects. That is, they exhibit a distinctive “numerosity look” or appearance. An intentionalist explanation of these appearances would be that numerosity looks obtain in virtue of some property, or properties, that these experiences represent. According to Burr and Ross (2008, 2012), for example, we are directly sensitive to the actual numerosity of visual arrays, with this property featuring in the representational content of experience. Another view, defended by Durgin (1995, 2008), holds that we are sensitive to, and so represent, only the size and density of such arrays, and are merely indirectly sensitive to numerosity which is not itself represented in experience. Since these two views ascribe different representational contents to visual experience, the question arises as to which, if either, of them identifies the properties that underpin numerosity looks.

O’Sullivan argues that this apparent representational difference cannot be settled empirically because there is no fact of the matter as to which property is being represented. Indeed, we might equally well posit representation of a disjunctive property whose disjuncts include both numerosity and density in order to explain the phenomenal character of such visual arrays. Against the rejoinder that precisely this disjunctive property is represented in visual experience, O’Sullivan argues that at this point “the specifically representational features of the posited token mental state are no longer doing any work” (this issue). Thus, we might equally claim that the subject directly registers the numerosity look of the array in a way that does not involve the tokening of representational content, as per enactivist or relationalist explanations of numerosity phenomena, since it is indeterminate what, if any, content is represented. This apparent indeterminacy surrounding the attribution of visual content raises doubts as to the adequacy of the representational view’s characterisation of the content of experience.

O’Sullivan’s argument highlights the difficulty in offering representationalist explanations of perceptual phenomena to support the position in cases where alternative non-representational explanations are also available. A representationalist might respond to this by questioning whether O’Sullivan’s account generalises to cover more ‘primitive’ properties of visual experience, such as shape or colour, or to other sensory modalities. Alternatively, they might argue that the alleged indeterminacy reflects an epistemological difficulty in determining what the relevant representational content is, rather than raising a deeper metaphysical question concerning its existence. Finally, they might simply agree that perceptual content need not straightforwardly map onto our pre-theoretical intuitions about individual cases, while retaining the idea that experiences are nevertheless representational. Each of these possibilities raises interesting methodological questions concerning precisely how perceptual content is to be individuated, and its explanatory role in grounding or justifying belief.

### J. A. Judge

As a rule to which this collection is no exception, philosophers of perception have tended to focus upon visual perception in the (possibly misguided) hope that theories of vision will generalise to cover all of the other sensory modalities which one might take humans to have. Moreover, whilst the existence and individuation of perceptual content have been widely discussed, the processes that generate and operate upon such contents have received much less attention. In a welcome departure from both of these unfortunate tendencies, J. A. Judge examines whether the auditory phenomenon known as the ‘missing fundamental’, first highlighted by Helmholtz (1954) and in which subjects are able to ‘hear’ the pitch of a musical note without its fundamental frequency actually being present, necessitates the existence of sub-personal perceptual inference. This might be argued to constitute evidence in favour of a representational view of auditory perception, since in order to perform such an inference the auditory system must presumably be capable of representing both the incoming stimuli and its inferred cause.

Inferentialist explanations are typically used to address the problem of the underdetermination of external stimuli (e.g. types of object) by proximal stimuli (e.g. retinal images). However, Judge argues that not only does the phenomenon of the ‘missing fundamental’ fail to constitute a clear candidate for the ascription of an inferential capacity, but that the notion of sub-personal inference is itself contentious and in need of further analysis. Following Boghossian (2008, 2014), Judge models sub-personal inference on rational inference, or rule-following. According to Boghossian, such cases involve both causal and representational constraints, since in order to count as following a particular rule, the rule itself must (i) be represented by the subject, and (ii) form part of a causal explanation of why they carry out the relevant inference. As Wittgenstein observed, however, merely acting in accordance with a given rule does not establish which rule, if any, is being followed. Thus, Judge argues, in order for the notion of sub-personal inference to have explanatory value in the theory of audition, the auditory system must make use of “internally stored knowledge” (Rock 1997: xiii), or representations of the rule to be followed. However, since the ‘missing fundamental’ is arguably neither a perceptual illusion nor a crucially impoverished stimulus, it does not appear to mandate this kind of explanation. Rather, as Judge puts it, “the system merely *looks* as though it is following a rule” (this issue).

Here, one might argue that the kind of inferences that are involved in auditory processing do not require the sort of explicit representation of rules that is present in the case of rational inference. If so, however, the burden of proof surely lies with the defenders of inferentialism to elucidate precisely what notion of inference is involved. Moreover, if such mechanisms are sub-personal, then it is not clear how they can constitute evidence that experiences are representational, since the relevant representations do not occur at the conscious level. Without some further theory connecting sub-personal representation to the content and/or phenomenal character of experience, the notion of sub-personal inference remains the preserve of neuroscientific and psychological explanations of perceptual processing rather than experience *per se*.

### Ivan V. Ivanov

In Chap. 2 of *The Contents of Visual Experience*, Susanna Siegel (2010a, b) presents an argument for the view that all visual experiences have contents — a claim she calls “the Content View” (CV). Siegel seeks to establish that the mere presentation of properties in visual experience entails that these experiences have accuracy conditions that are conveyed to the subject. Since Siegel defines the notion of content precisely in terms of such accuracy conditions, this in turn establishes CV. Siegel’s “Argument from Appearing” is intended to be neutral on the underlying metaphysics of experience, and so apply equally to both representational and relational views of experience. Thus, Siegel aims to establish the existence of a weak content view on the basis of an alleged phenomenological datum about visual experience: namely, that it involves the presentation of properties “as being instantiated” (Siegel 2010a: 45).

Ivan V. Ivanov (this issue) agrees with Siegel that it is plausible that visual experience consists in property-awareness rather than pure object-awareness, but takes issue with the alleged metaphysical neutrality of Siegel’s characterisation of properties. According to Ivanov, on both universalist and nominalist views of the metaphysics of property-types, a version of Siegel’s argument goes through. However, on the equivalent trope-theoretic formulation of relationalism, visual experience does not consist in the presentation of property-types, but abstract particulars, or tropes, where the presentation of a given particular does not convey to the subject the type of which it is an instance. Thus, rather than being aware of a universal, or fact, perceivers of an object that presents some property *F* are instead aware of *the object’s F-ness* or, equivalently, *the object’s being F*. By adopting tropism, Ivanov argues that the relationalist can consistently endorse the seemingly plausible idea that experiences ‘present properties’ while rejecting Siegel’s representationalist conclusion.

Ivanov’s analysis highlights two important distinctions that arise from Siegel’s argument. First, the distinction between the presentation of *property-types*, or universals, and the presentation of *property-instances*, e.g. tropes (Ivanov considers non-tropist versions of the latter view in the final section of his paper). If Siegel’s argument depends upon the first notion of presentation, then it begs the question against relationalists like Travis (2004, 2013a) who think that visual experience involves only the presentation of particulars, and not general types. The presentation of property-instances, on the other hand, is more in keeping with relationalists’ characterisation of experience. However, Ivanov argues, on a trope-theoretic understanding of property-instances, this need not yield accuracy conditions for experience that are conveyed to the subject in the way that Siegel’s argument requires.

The second distinction concerns the difference between what we might call the *mere presentation* of objects or properties, i.e. what is actually presented to the subject, and these being *presented as of* *being some particular way*—an intentional notion. While relationalists agree that experiences are presentational in the first sense, they typically deny this entails that they are presentational in the second sense, except insofar as the phenomenal character of those experiences is partly constituted by the particular objects or properties that are in fact presented. Ivanov argues that even Siegel’s minimal characterisation of the latter notion of presentation fails to remain neutral on the metaphysics of experience, thereby begging the question in favour of CV and undermining the force of the Argument from Appearing. Whether relationalists can consistently endorse the extensional notion of property presentation while also denying that objects are presented as of instantiating some relevant property-type, however, remains a central question in the debate.

## Future Directions

As we hope this collection demonstrates, whether perceptual experience is representational or otherwise goes well beyond the question of consistency with perceptual science or adherence to ‘common-sense’ assumptions about perception. Indeed, what is at issue in many of these debates is not so much the existence of perceptual representation, but its explanatory role, or roles, within a philosophically adequate theory of perception. As such, hard-line anti-representationalists like Travis (2004, 2013a) dispute that any such content could satisfy what they take to be the conflicting demands of explaining the variability of perceptual appearances and grounding rational judgements about external mind-independent objects. Even so, more moderate relationalists like Brewer, however, who deny that such contents features in the most fundamental characterisation of perceptual experience, still find a role for representation in explaining aspects of perceptual phenomenology such as conceptual registration or aspect-switching—a conclusion that is echoed by Brogaard (this issue). Furthermore, while genuine attempts to reconcile the divide between representational and relational views exist (e.g. Schellenberg 2011, 2016; Logue 2014), given the wide variety of explanatory demands that representationalists place upon perceptual content, the precise shape that such hybrid theories should take remains an open and interesting question.

Conversely, given the strongly externalist nature of many contemporary theories of perceptual content, one might be forgiven for thinking that representationalists have already incorporated many of the insights of relationalism, such as the particularity of perception, or its dependence upon environmental factors, into their view. However, if we take representationalism to offer a theory of the content of *conscious perceptual experiences*, and not merely the sub-personal mechanisms involved in perceptual processing, the choice appears less clear cut. To establish this stronger role for representation it is necessary not only to show that experiences possess contents, or have accuracy conditions, but that these contents are in some way manifest, or cognitively available, to the perceiving subject at a first-personal level. This in turn highlights the distinction between the ‘mere’ presentation of external objects and their properties in perception, and those objects being presented, or represented, as of being some particular way, e.g. as instantiating some general type, where only the latter entails the existence of propositional or intentional content. While alternative non-propositional notions of content are available (Crane 1992, 2009), it falls to their proponents to explain why we should invoke the notion of *representation* in the explanation of perceptual experience, as opposed to an analogous non-representational form of explanation, assuming that one is available.

A further question that is raised by several of the following essays concerns the relation between the philosophy of mind and perception on the one hand, and broader metaphysical issues, such as the nature of relations or properties, on the other. While the nature of perceptual experience is itself a metaphysical issue, the extent to which this dispute turns on further metaphysical and ontological commitments outside the philosophy of mind has perhaps been underestimated. Just as varying the referents of perceptual representations or the relata of the perceptual relation has the potential to yield quite different theories of perception, whether reality consists of objects, universals or tropes may have significant implications for theories of perceptual awareness. Clarifying the metaphysical commitments of both relational and representational views of experience, including the precise explanatory role (or roles) of perceptual representation, the nature of the perceptual relation, and the sense in which external particulars may be said to ‘constitute’ an experience’s phenomenal character, thus remain central and important tasks for contemporary philosophy of perception.

## Footnotes

1 For brevity, we will use ‘experience’ to mean all conscious perceptual episodes, where this includes both (i) veridical perception and (ii) non-veridical perceptual illusions and hallucinations.

2 We use the capitalised term to refer to the philosophical theory, and to differentiate it from ‘direct realism’ (see Sects. 2 and 4).

3 Among early proponents of sense-data the nature of these entities was an open question. Moore (1926), for example, takes sense-data to be parts of the surfaces of objects. After lengthy discussion, Price (1932) concludes that sense-data are not physical, but mind-independent, while Russell (1921) argues that they are neither physical nor mental. In recent discussions the assumption that sense-data are non-physical or mind-dependent entities that resemble or stand in some external (e.g. causal) relation to ordinary physical objects has prevailed (see Robinson 1994; Foster 2000; Huemer 2011).

4 The *locus classicus* for such objections is Barnes (1944). Contemporary defenders of sense-datum theories include Jackson (1977), Robinson (1994), and Foster (2000).

5 For example, many philosophers claim that there are irreducible qualitative properties, or *qualia*, that either totally (e.g. Block 2003, 2010) or in part (e.g. Peacocke 1983; Block 1996; Shoemaker 1990) determine the phenomenal character of experience, though we do not engage directly with these views here.

6 See also Crane (1998) and Kriegel (2016).

7 For a survey of recent work, see Brogaard (2014b), Hawley and Macpherson (2011), and Siegel (2016).

8 For a discussion of the various kinds of veridicality, see Siegel (2010a, b).

9 See Travis (2004, 2013a), Siegel (2010a, b), Wilson (forthcoming) and Ivanov (this issue).

10 See Sect. 4, Martin (this issue), and Travis (this issue).

11 Unfortunately, neither term is used consistently throughout the literature, with some authors failing to distinguish between the two, or using ‘representationalism’ and ‘intentionalism’ more or less interchangeably. To avoid confusion, we will adopt the terms ‘representational view(s)’ or ‘representationalism’ for representational theories in general, while reserving ‘intentionalism’ for claims that relate specifically to phenomenal character. Any divergences from this usage are noted below. For further discussion of intentionalism, see Byrne (2001) and Crane (2009).

12
*Mutatis mutandis* for views that appeal to manners of representation.

13 Logue’s “Mild Content View” (2014: 223).

14 Travis (2013c: 3), for example, describes representation as a three-place relation.

15 This list is by no means exhaustive. For further discussion, see Soteriou (2014, 2016).

16 Precisely how we should understand this claim is an important issue, though not one we have space to fully engage with here.

17 E.g. Byrne (2009). For discussion of forms of relationalism that treat illusions as being akin to hallucinations, see Byrne and Logue (2008).

18 A claim that is disputed by Burge (2005, 2011).

19 Price (1932) discusses at length what he calls ‘Selective Theory’ which endorses a relational view of perception and a sense-datum view of hallucination, where sense data are mind-independent non-physical objects. Austin (based on remarks in his 1962: 50, 52, 54) may be read as suggesting, at least for the sake of argument, a form of disjunctivism of this kind (see Byrne and Logue 2008; Soteriou 2016: 18 for this interpretation of Austin). Langsam (1997) maintains that in hallucination we are aware of a portion of space. To our knowledge, no one has yet combined a relational account of perception with an intentional account of hallucination.

20 For an overview, see Crane and French (2016: Sect. 3.2).

21 For further discussion of the distinction between direct and indirect realism, see Snowdon (1992), and Crane and French (2016).

22 Travis’s notion of the “conceptual” includes representations of particular objects and events that involve concepts which are satisfiable by just one historical individual.

23 Notably, Travis does not rule out the existence of sub-personal representations—as part of a neuroscientific explanation of perceptual processing, for example

24 For alternative formulations of, and responses to, Travis’s argument, see Brogaard (this issue) and Raleigh (2013).

25 Brogaard uses “Representational View” to mean what we have called ‘intentionalism’ (Sect. 2).

26 One might resist this claim on the basis of the cognitive penetration of experience by belief (Stokes 2013) or there being no sharp distinction between perception and cognition, as in predictive processing models (Clark 2016).

27 Though Brewer focuses exclusively on vision, his account is presumably intended to generalise to other sensory modalities.

28 Campbell here assumes that the representationalist is committed to conceptual content, though his arguments for this claim remain largely implicit.
